# Simultaneous Presentation of Post-Transplant Lymphoproliferative Disorder (PTLD) and Acute Cellular Rejection (ACR) in a Liver Transplant Recipient: A Therapeutic Conundrum

**DOI:** 10.1155/crgm/1627234

**Published:** 2025-10-29

**Authors:** Ankit Mishra, Matthew Kubina, Dhiraj K. Peddu, Benjamin L. Viglianti, Anamarija M. Perry, Priya Kathuria, Hellan Kwon, Shannon A. Carty, Patricia Bloom

**Affiliations:** ^1^Department of Internal Medicine, University of Michigan, Ann Arbor, Michigan, USA; ^2^Division of Nuclear Medicine and Molecular Imaging, Department of Radiology, University of Michigan, Ann Arbor, Michigan, USA; ^3^Department of Pathology, University of Michigan, Ann Arbor, Michigan, USA; ^4^Division of Gastroenterology and Hepatology, Henry Ford Hospital, Detroit, Michigan, USA; ^5^Division of Gastroenterology and Hepatology, University of Michigan, Ann Arbor, Michigan, USA; ^6^Division of Hematology and Oncology, Rogel Cancer Center, University of Michigan, Ann Arbor, Michigan, USA

## Abstract

We report a 64-year-old liver transplant recipient who developed early nondestructive post-transplant lymphoproliferative disorder (PTLD) and severe acute cellular rejection (ACR) concurrently. Hepatic lymphadenopathy led to a liver biopsy demonstrating early PTLD. Immunosuppression (IS) was reduced for early PTLD, which led to acute liver injury requiring high-dose steroids. However, subsequent augmentation in immunosuppression for ACR led to progression of PTLD, requiring rituximab treatment. This case highlights the complexity of managing conflicting liver transplant complications and underscores the importance of a multidisciplinary approach. In our case, prioritizing the treatment of rejection preserved the allograft function. Long-term follow-up showed complete resolution of both rejection and PTLD.

## 1. Introduction

Post-transplant lymphoproliferative disorder (PTLD) and severe acute cellular rejection (ACR) are post liver transplantation (LT) complications, each contributing to high rates of morbidity and mortality, and necessitate divergent treatment strategies. PTLD occurs through uncontrolled lymphocyte proliferation, with early PTLD typically driven by Epstein–Barr virus (EBV) [1, 2]. ACR is also common after LT, with an incidence of up to 25%–30% and often occurring within 1 month post-transplantation [3]. ACR occurs due to recipient T cells recognizing donor alloantigens, which results in T-cell recruitment and an innate immune response [4]. The mainstay of early, nondestructive PTLD management is reduction of immunosuppression, while ACR is usually managed by augmenting IS, typically with high-dose steroids and/or escalation in immunosuppressive therapies [3]. Specifically, recommendations state to discontinue antimetabolites (e.g., azathioprine and mycophenolate mofetil) and reduce calcineurin inhibitors by 50%; however, such reduction carries a risk of rejection [5]. Our literature search did not identify any liver transplant cases with biopsy-proven concurrent PTLD and ACR, but a case report published in 2001 demonstrated a similar clinical scenario in a lung transplant patient [6]. This patient likely had coexistent ACR and PTLD which is different from our case where the patient developed ACR after RIS for early PTLD [6]. The authors in this study point out the possibility of two different subpopulations of T cells: EBV-specific T cells implicated in PTLD and allospecific T cells directed toward alloantigens in ACR [6]. This report describes the first documented instance of simultaneously managing PTLD and severe acute liver rejection, underscoring the necessity of a tailored, multidisciplinary approach.

## 2. Case Report

A 64-year-old man with a history of Type 2 diabetes mellitus, hypertension, metabolic dysfunction–associated steatotic liver disease with cirrhosis, and multifocal moderately differentiated hepatocellular carcinoma (HCC), status-post orthotopic LT (donor EBV+, recipient EBV-) in August 2022, underwent a routine liver MRI on postoperative day (POD) 279 as part of the intermediate risk protocol for HCC monitoring. The MRI revealed new porta hepatis lymphadenopathy. Two porta hepatis lymph nodes biopsies obtained via endoscopic ultrasound on POD 377 and 438, respectively, were pathologically benign. A follow-up MRI on POD 445 showed continued enlarging perihepatic lymph nodes. Given the continued enlarging lymph nodes on liver MRI, the patient underwent a liver biopsy on POD 481.

The liver biopsy demonstrated few aggregates of small lymphocytes and scattered EBV-positive cells, with a quantitative plasma EBV (qEBV) level of 42,882 I.U./mL. Given the nondisruptive nature of lymphocytes, as well as elevated serum EBV viral load, there was a concern for early PTLD, and the patient's cyclosporine dose was reduced from 100 to 75 mg twice daily on POD 488. On POD 519, the patient was urgently admitted to the inpatient liver service at the University of Michigan for rising liver biochemistries, consistent with mixed pattern of injury in the setting of recently decreased immunosuppression (aspartate aminotransferase 93–321 U/L [< 34]; alanine aminotransferase 117–420 U/L [10–49]; alkaline phosphatase 187–287 U/L [40–116]; and total bilirubin 0.8–1.1 mg/dL [0.2–1.2]).

**Figure 1:**
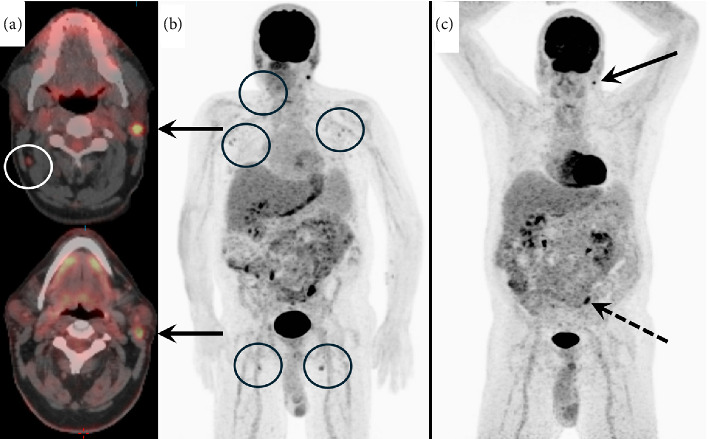
Pre- and postrituximab therapy images. Patient in (a) axial PET CT images of the neck prior to therapy (top) and post-therapy (bottom). In the pretherapy image, a right Level 2 lymph node (white circle) demonstrated pathological uptake. In (b), maximal intensity projection (MIP) image of the FDG PET data demonstrates abnormal nodal uptake above and below the diaphragm (black circles). In (c), post-therapy MIP image are shown with resolution of nodal uptake. Persistent uptake in the left parotid gland (black arrow) and the left abdomen/colon (dashed arrow) is shown. The left parotid is consistent with Warthin's tumor, and the colonic uptake is an adenoma confirmed by colonoscopy.

**Figure 2:**
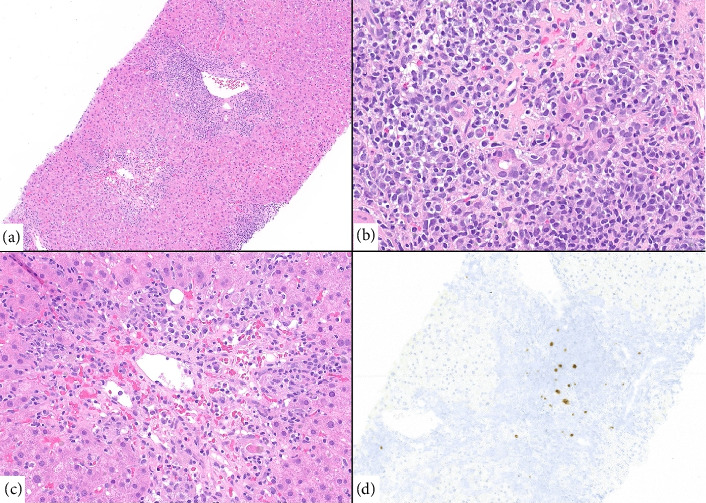
Liver with acute cellular rejection and increased EBV-positive cells. (a) Lower magnification of liver biopsy showing densely inflamed portal tract and central vein with pericentral hepatocyte dropout. (b) High-power magnification of the portal tract shows mixed inflammatory infiltrate with numerous plasma cells and bile duct injury by lymphocytes. (c) Centrilobular area shows lack of hepatocytes and endotheliitis (Banff 7). (d) EBER in situ hybridization highlights increased EBV-positive cells in the portal tract.

**Figure 3:**
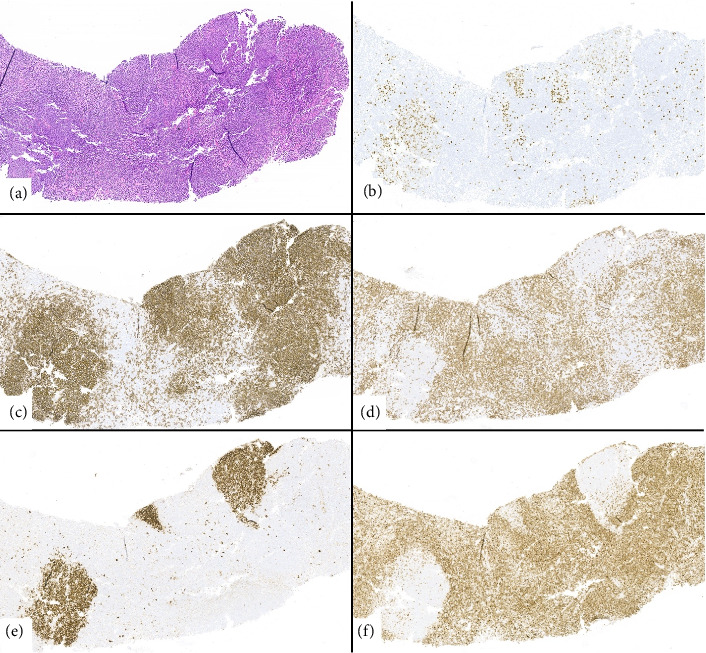
Lymph node involved by early post-transplantation lymphoproliferative disorder (PTLD). (a) Lymph node shows preserved architecture with follicular hyperplasia. (b) EBER in situ hybridization shows numerous EBV-positive cells in reactive follicles and paracortical area. (c) CD20 and (d) CD30 highlight B- and T-cell areas, respectively. (e) CD10 stains reactive germinal centers which are negative for (f) BCL2.

During this admission, a nuclear medicine fluorodeoxyglucose (FDG) positron emission tomography (PET) scan showed FDG-avid nonspecific lymphadenopathy in the thorax, abdomen, and pelvis (Figures 1(a), 1(b)). The patient's qEBV level was 2518 I.U./mL. A cervical lymph node and liver biopsy were performed. The liver biopsy indicated a severe bile duct injury and endotheliitis concerning for severe ACR (Figure 2). The cervical lymph node biopsy showed follicular hyperplasia with numerous EBV-positive cells, consistent with early/non-destructive PTLD (Figure 3). After extensive discussion with pathology, hematology, and hepatology, the patient was treated with a three-day course of methyl-prednisolone (100 mg on Day 1, 250 mg on Day 2, and 250 mg on Day 3) for severe acute cellular liver rejection. This was deemed the most immediate threat to preserve his transplanted liver function [7].

**Figure 4:**
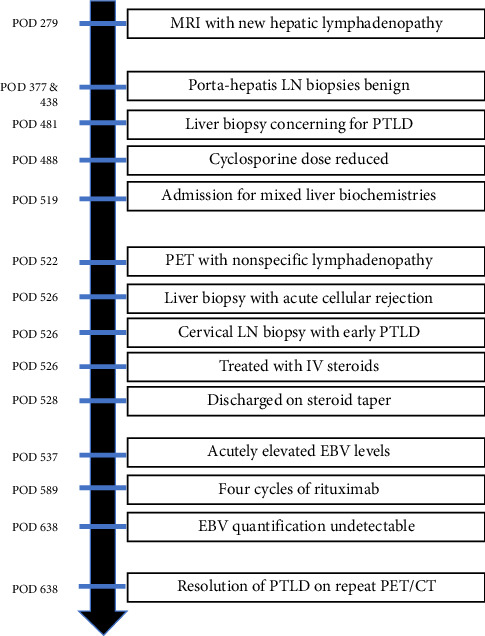
Chronological overview of events throughout the patient's clinical course. POD = postoperative day.

Shortly after his treatment with high-dose IV steroids, he was discharged home with a steroid taper (60 mg for 7 days, 50 mg for 7 days, and then 40 mg for 7 days with ongoing prednisone taper) and his prior IS regimen (mycophenolate mofetil 500 mg two times daily and cyclosporine 75 mg two times daily). A few days later, he underwent outpatient laboratory testing to monitor his liver function tests (LFTs) as well as qEBV levels. This demonstrated improving liver biochemistries, but acute elevation in EBV level from 2518 to 10,252 I.U./mL, concerning for rapidly progressing PTLD. The patient was evaluated by hematology and received four doses of weekly rituximab 375 mg/m^2^ for early PTLD in the setting of ACR preventing a reduction in immunosuppression. Post-treatment, his EBV quantification rapidly came down to undetectable (10,252 I.U./mL ⟶ 211 I.U./mL ⟶ 59 I.U./mL ⟶ < 35 I.U./mL ⟶ undetectable), PET/CT demonstrated complete metabolic response, and his LFTs have remained within normal limits (Figure 1(c)). The patient had no recurrence of PTLD or ACR with the last follow-up on POD 932. He has remained on prednisone 3 mg daily, mycophenolate mofetil 500 mg two times daily, and cyclosporine 75 mg two times daily (last trough on 8/26/25 was 99 ng/mL) (Figure 4).

## 3. Discussion

ACR and PTLD are distinct pathophysiological processes, each requiring careful management in transplant recipients. Lifelong immunosuppression is essential for all transplant patients to preserve liver allograft function. PTLD is a serious post-transplant complication, occurring in 1%–20% of solid organ transplant (SOT) recipients [1, 8]. Based on timing, PTLD is classified into early (12–24 months post-SOT) and late (> 24 months post-SOT) categories [8]. Additionally, PTLD is further categorized into nondestructive, polymorphic, and monomorphic subtypes. First-line treatment for early nondestructive PTLD typically involves reducing immunosuppression, enabling the patient's immune system to mount a T-cell response against proliferating malignant cells [9]. In contrast, polymorphic and monomorphic PTLD often require reduction in immunosuppression, rituximab, and sometimes immunochemotherapy, in cases of B-cell lymphoid neoplasms [8].

Rejection occurs when the recipient's immune system mounts a response against the donor's major histocompatibility complex [10]. The Banff Working Group classifies cellular rejection as T-cell-mediated rejection (TCMR) with acute and chronic components [11, 12]. TCMR is primarily defined by T-cell infiltrates, with histologic features including ductulitis, portal or central venous endotheliitis, and mixed portal inflammation [10, 13]. Notably, minimal to no inflammatory activity is observed in hepatocytes adjacent to the portal triad's connective tissue [13].

Our patient presented with an unusual case of early PTLD alongside features suspicious for severe ACR. To date, no documented cases have demonstrated the concurrent presentation of these two distinct pathophysiological processes. The liver showed plasmacytic infiltrates, atypical lymphocytes, and severe bile duct injury with endotheliitis (Figure 2), findings suggestive of severe ACR. The Banff score for this patient's ACR was 7, with individual scores of 3 for portal inflammation, 3 for bile duct damage, and 1 for venous endothelial inflammation.

The cervical lymph node met the histologic criteria for early PTLD; however, the histologic appearance of the liver infiltrates did not align with PTLD, as there was no morphologic evidence of tissue effacement (Figure 3). We believe that the EBV-positive cells in the liver were likely due to EBV viremia. Rituximab therapy carries important infection risks, particularly when used with corticosteroids. These include hepatitis B virus reactivation and *pneumocystis jirovecii* pneumonia (PJP) [14, 15]. For our patient, we began trimethoprim-sulfamethoxazole (400/80 mg once daily) when pulse dose steroids were started for PJP prophylaxis and obtained HBV serologies prior to initiating rituximab.

In summary, we present this unique case to highlight the clinical dilemma where both increased and decreased IS are required simultaneously [7]. In this specific scenario, high-dose steroids followed by one course of weekly rituximab successfully managed both conditions. The uniqueness of this case lies in the fact that while reducing immunosuppression is typically sufficient for early nondestructive PTLD, it was not a viable option due to the presence of severe ACR.
